# Upregulation of dNTP Levels After Telomerase Inactivation Influences Telomerase-Independent Telomere Maintenance Pathway Choice in *Saccharomyces cerevisiae*

**DOI:** 10.1534/g3.118.200280

**Published:** 2018-05-30

**Authors:** Paula M. van Mourik, Jannie de Jong, Sushma Sharma, Alan Kavšek, Andrei Chabes, Michael Chang

**Affiliations:** * European Research Institute for the Biology of Ageing, University of Groningen, University Medical Center Groningen, 9713 AV Groningen, the Netherlands; † Department of Medical Biochemistry and Biophysics; ‡ Laboratory for Molecular Infection Medicine Sweden (MIMS), Umeå University, SE 901 87 Umeå, Sweden

**Keywords:** *Saccharomyces cerevisiae*, telomeres, telomerase-independent telomere maintenance, survivors, dNTP levels

## Abstract

In 10–15% of cancers, telomere length is maintained by a telomerase-independent, recombination-mediated pathway called alternative lengthening of telomeres (ALT). ALT mechanisms were first seen, and have been best studied, in telomerase-null *Saccharomyces cerevisiae* cells called “survivors”. There are two main types of survivors. Type I survivors amplify Y′ subtelomeric elements while type II survivors, similar to the majority of human ALT cells, amplify the terminal telomeric repeats. Both types of survivors require Rad52, a key homologous recombination protein, and Pol32, a non-essential subunit of DNA polymerase δ. A number of additional proteins have been reported to be important for either type I or type II survivor formation, but it is still unclear how these two pathways maintain telomeres. In this study, we performed a genome-wide screen to identify novel genes that are important for the formation of type II ALT-like survivors. We identified 23 genes that disrupt type II survivor formation when deleted. 17 of these genes had not been previously reported to do so. Several of these genes (*DUN1*, *CCR4*, and *MOT2*) are known to be involved in the regulation of dNTP levels. We find that dNTP levels are elevated early after telomerase inactivation and that this increase favors the formation of type II survivors.

Eukaryotic chromosomes have specialized structures at their termini called telomeres. Telomeres prevent natural chromosome ends from being recognized and processed as DNA double-strand breaks in need of repair (Jain and Cooper 2010). Due to incomplete DNA replication and nucleolytic degradation, telomeres shorten with each round of cell division. Telomere shortening is reversed by the action of telomerase, a specialized reverse transcriptase that extends telomeres (Greider and Blackburn 1985). However, most human somatic cells do not express sufficient levels of telomerase to prevent telomere shortening, which has been implicated in human aging (López-Otin *et al.* 2013). The downregulation of telomerase early during human development has been proposed to function as a barrier to tumorigenesis because cancers cells need to maintain their telomeres to avoid replicative senescence or apoptosis induced by telomere erosion (Hanahan and Weinberg 2011). Most cancer cells overcome this barrier by reactivating telomerase, but 10–15% of cancers employ a telomerase-independent pathway known as alternative lengthening of telomeres (ALT) (Sobinoff and Pickett 2017).

In the budding yeast *Saccharomyces cerevisiae*, telomerase is constitutively expressed, allowing the maintenance of telomeres 300 ± 75 bp in length (Wellinger and Zakian 2012). The core components of telomerase in *S. cerevisiae* are a protein catalytic component (Est2) and an RNA subunit (TLC1) (Lingner *et al.* 1997; Singer and Gottschling 1994). Abrogating telomerase function, for example by deleting either *EST2* or *TLC1*, will cause telomere attrition and, eventually, cell cycle arrest and replicative senescence. A small subset of cells can overcome senescence and become what are called “survivors” (Lundblad and Blackburn 1993), using telomerase-independent telomere maintenance mechanisms as in ALT cancer cells.

There are two main types of *S. cerevisiae* survivors: type I and type II. Type I survivors exhibit amplification of the subtelomeric Y′ elements; in contrast, type II survivors amplify the terminal (TG_1-3_)_n_ telomeric sequences (Lundblad and Blackburn 1993; Teng and Zakian 1999). Type I and type II survivors require Rad52-dependent homologous recombination (HR) and the DNA polymerase δ subunit Pol32, which is required for break-induced replication (BIR), suggesting that both survivor pathways occur through recombination-dependent DNA replication (Lundblad and Blackburn 1993; Lydeard *et al.* 2007). The Pif1 helicase is also important for the generation of type I and type II survivors (Dewar and Lydall 2010), likely due to its role in BIR (Saini *et al.* 2013; Wilson *et al.* 2013). There are two BIR pathways: one is Rad51-dependent and one is independent of Rad51, but requires the MRX complex (consisting of Mre11, Rad50, and Xrs2) and Rad59 (Anand *et al.* 2013). Similarly, the formation of type I survivors is dependent on Rad51 (and Rad54 and Rad57, which function in the same pathway as Rad51), whereas type II survivors require the MRX complex and Rad59 (Teng *et al.* 2000; Chen *et al.* 2001), suggesting that type I and type II survivors maintain telomeres via Rad51-dependent and Rad51-independent BIR, respectively.

Type II survivors resemble the majority of human ALT cells in that both are characterized by long and heterogeneous-sized telomere length (Teng and Zakian 1999; Bryan *et al.* 1995; Bryan *et al.* 1997), extrachromosomal circular DNA containing telomeric sequence (Larrivée and Wellinger 2006; Cesare and Griffith 2004; Henson *et al.* 2009), and telomere maintenance by Rad51-independent BIR requiring the MRX (or MRN—Mre11, Rad50, Nbs1—in humans) complex (Teng *et al.* 2000; Chen *et al.* 2001; Dilley *et al.* 2016; Jiang *et al.* 2005; Zhong *et al.* 2007).

Sgs1 and Exo1, which are needed for processive resection of DNA ends (Mimitou and Symington 2008; Zhu *et al.* 2008), are also important for type II survivor formation (Huang *et al.* 2001; Johnson *et al.* 2001; Maringele and Lydall 2004; Bertuch and Lundblad 2004). Consistent with the importance of end resection for type II survivor formation, the *sgs1-D664∆* mutation (Bernstein *et al.* 2009; Bernstein *et al.* 2013), which is competent for recombination repair but defective in resection, also prevents the formation of type II survivors (Hardy *et al.* 2014). Similarly, type II survivor formation is hindered by the deletion of *FUN30*, which encodes a chromatin remodeler that promotes end resection (Costelloe *et al.* 2012). BLM, a human homolog of Sgs1, has also been implicated in facilitating telomere maintenance in ALT cells (Stavropoulos *et al.* 2002).

Several additional proteins have also been implicated in the formation of type II survivors. These include the Tel1 and Mec1 DNA damage checkpoint kinases: in the absence of either Mec1 or Tel1, type II survivor formation is impaired, and is completely abolished in *mec1Δ tel1Δ* double mutants (Tsai *et al.* 2002). Furthermore, the RNA polymerase II degradation factor Def1, the B-type cyclin Clb2, the tRNA modification protein Sua5, and Mdt4/Pin4, which interacts with the DNA damage kinase Rad53, are also important for type II survivor formation (Chen *et al.* 2005; Grandin and Charbonneau 2003; Meng *et al.* 2010; Pike and Heierhorst 2007). An analysis of 280 genes known to alter telomere length homeostasis when deleted further identified 22 genes that are important for type II survivor formation, including genes encoding members of the nonsense mediated decay pathway, the DNA repair protein Rad6, and the KEOPS complex (Hu *et al.* 2013). However, it is still unclear how most of these proteins function in the formation of type II survivors, and whether there are more proteins involved in this process.

In this study, we performed a genome-wide screen to identify novel genes that are important for the formation of type II survivors. We identified 23 genes, 17 of which were not previously reported to be involved in type II survivor formation. Several of these genes are involved in the regulation of intracellular deoxyribonucleoside triphosphate (dNTP) levels. We show that dNTP levels are increased early after inactivation of telomerase, and that this increase is important to generate type II survivors.

## Materials and Methods

### Yeast strains and growth conditions

Standard yeast media and growth conditions were used (Treco and Lundblad 2001; Sherman 2002). With the exception of MCY610 and the yeast knockout (YKO) collection (Giaever *et al.* 2002), all yeast strains used in this study are *RAD5* derivatives of W303 (Thomas and Rothstein 1989; Zhao *et al.* 1998) and are listed in Table 1. MCY610 has a hybrid BY4741 and W303 genetic background. Generation of survivors on agar plates and in liquid culture was performed as previously described (van Mourik *et al.* 2016).

**Table 1:** Yeast strains used in this study

Strain name	Relevant genotype	Source
MCY610	*MAT***a**/α *can1∆STE2pr-HIS3/can1∆STE2pr-Sp_his5 lyp1∆/lyp1∆ rad51ΔURA3 /RAD51 est2∆natMX/EST2 TRP1/trp1-1 ADE2/ADE2 his3∆1/his3 leu2∆0/leu2 ura3∆0/ura3 RAD5/rad5-535*	This study
CCY6	*MAT***a**/α *est2ΔURA3/EST2*	Clémence Claussin
CCY16	*MAT***a**/α *est2ΔURA3/EST2 rad52ΔnatMX/RAD52*	Claussin and Chang 2016
YPM7	*MAT***a**/α *est2ΔURA3/EST2 rad51ΔnatMX/RAD51 rad50ΔkanMX/RAD50*	This study
YPM8	*MAT***a**/α *est2ΔURA3/EST2 rad51ΔnatMX/RAD51 rad59ΔkanMX/RAD59*	This study
YPM9	*MAT***a**/α *est2ΔURA3/EST2 rad51ΔnatMX/RAD51*	This study
YPM10	*MAT***a**/α *est2ΔURA3/EST2 rad51ΔnatMX/RAD51 nmd2ΔkanMX/NMD2*	This study
YPM11	*MAT***a**/α *est2ΔURA3/EST2 rad51ΔnatMX/RAD51 rgi1ΔkanMX/RGI1*	This study
YPM12	*MAT***a**/α *est2ΔURA3/EST2 rad51ΔnatMX/RAD51 dun1ΔTRP1/DUN1 sml1ΔHIS3/SML1*	This study
YPM17	*MAT***a**/α *est2ΔURA3/EST2 rad51ΔnatMX/RAD51 clb2ΔkanMX/CLB2*	This study
YPM20	*MAT***a**/α *est2ΔURA3/EST2 rad51ΔnatMX/RAD51 vps25ΔkanMX/VPS25*	This study
YPM21	*MAT***a**/α *est2ΔURA3/EST2 rad51ΔnatMX/RAD51 lsm1ΔkanMX/LSM1*	This study
YPM29	*MAT***a**/α *est2ΔURA3/EST2 rad51ΔnatMX/RAD51 rmi1ΔkanMX/RMI1*	This study
YPM30	*MAT***a**/α *est2ΔURA3/EST2 rad51ΔnatMX/RAD51 spt20ΔkanMX/SPT20*	This study
YPM31	*MAT***a**/α *est2ΔURA3/EST2 rad51ΔnatMX/RAD51 cdc55ΔkanMX/CDC55*	This study
YPM32	*MAT***a**/α *est2ΔURA3/EST2 rad51ΔnatMX/RAD51 chk1ΔkanMX/CHK1*	This study
YPM33	*MAT***a**/α *est2ΔURA3/EST2 rad51ΔnatMX/RAD51 pph3ΔkanMX/PPH3*	This study
YPM34	*MAT***a**/α *est2ΔURA3/EST2 rad51ΔnatMX/RAD51 mot2ΔkanMX/MOT2*	This study
YPM35	*MAT***a**/α *est2ΔURA3/EST2 rad51ΔnatMX/RAD51 rpn4ΔkanMX/RPN4*	This study
YPM36	*MAT***a**/α *est2ΔURA3/EST2 rad51ΔnatMX/RAD51 ylr358cΔkanMX/YLR358C*	This study
YPM37	*MAT***a**/α *est2ΔURA3/EST2 rad51ΔnatMX/RAD51 rrm3ΔkanMX/RRM3*	This study
YPM38	*MAT***a**/α *est2ΔURA3/EST2 rad51ΔnatMX/RAD51 tsc3ΔkanMX/TSC3*	This study
YPM39	*MAT***a**/α *est2ΔURA3/EST2 rad51ΔnatMX/RAD51 pxp1ΔkanMX/PXP1*	This study
YPM40	*MAT***a**/α *est2ΔURA3/EST2 rad51ΔnatMX/RAD51 mtc7ΔkanMX/MTC7*	This study
YPM41	*MAT***a**/α *est2ΔURA3/EST2 rad51ΔnatMX/RAD51 doa4ΔkanMX/DOA4*	This study
YPM42	*MAT***a**/α *est2ΔURA3/EST2 rad51ΔnatMX/RAD51 cik1ΔkanMX/CIK1*	This study
YPM43	*MAT***a**/α *est2ΔURA3/EST2 rad51ΔnatMX/RAD51 ure2ΔkanMX/URE2*	This study
YPM44	*MAT***a**/α *est2ΔURA3/EST2 rad51ΔnatMX/RAD51 vma22ΔkanMX/VMA22*	This study
YPM45	*MAT***a**/α *est2ΔURA3/EST2 rad51ΔnatMX/RAD51 rpl8bΔkanMX/RPL8B*	This study
YPM48	*MAT***a**/α *est2ΔURA3/EST2 rad51ΔnatMX/RAD51 ylr235cΔkanMX/YLR235C*	This study
YPM51	*MAT***a**/α *est2ΔURA3/EST2 rad51ΔnatMX/RAD51 ccr4ΔkanMX/CCR4*	This study
YPM55	*MAT*α *est2ΔURA3* type II survivor	This study
YPM56	*MAT*α *est2ΔURA3* type II survivor	This study
MCY775	*MAT***a**/α *est2ΔURA3/EST2 dun1ΔTRP1/DUN1 sml1ΔHIS3/SML1*	This study
MCY783	*MAT***a** *est2ΔURA3* type II survivor	This study
MCY784	*MAT***a** *est2ΔURA3* type II survivor	This study
MCY785	*MAT***a** *est2ΔURA3 sml1∆HIS3* type II survivor	This study
MCY786	*MAT***a** *est2ΔURA3 sml1∆HIS3* type II survivor	This study
MCY788	*MAT***a** *est2ΔURA3 dun1∆TRP1 sml1∆HIS3* type II survivor	This study
YPM60	*MAT***a** *est2ΔURA3* type II survivor	This study
YPM61	*MAT***a** *est2ΔURA3 dun1∆TRP1* type II survivor	This study
YPM62	*MAT***a** *est2ΔURA3 dun1∆TRP1* type II survivor	This study
YPM63	*MAT***a** *est2ΔURA3 dun1∆TRP1* type II survivor	This study
YPM64	*MAT***a** *est2ΔURA3 dun1∆TRP1 sml1∆HIS3* type II survivor	This study
YPM65	*MAT***a** *est2ΔURA3 dun1∆TRP1 sml1∆HIS3* type II survivor	This study

### SGA screening procedure

The *est2∆* and *rad51∆* deletions were introduced into the strains of the YKO collection using synthetic genetic array (SGA) methodology (Tong and Boone 2006). The *MAT*α *can1∆STE2pr-Sp_his5est2∆natMX his3leu2lyp1∆ RAD5rad51ΔURA3TRP1ura3* query strain for the screen was derived from the sporulation of MCY610. The pinning steps were performed using a ROTOR HDA (Singer Instruments, Somerset, UK) with a 384-density format. The final *est2∆natMX rad51∆URA3 xxx∆kanMX* triple mutants (where *xxx∆kanMX* represents a deletion of a gene from the YKO collection) were quadruplicated (*i.e.*, the plate density was increased to 1536), and the resulting four colonies per strain were individually streaked on YPD plates, followed by incubation at 30° for 3 days. The strains were re-streaked 5-6 times until senescence was observed and survivors were formed, or until senescence was observed but no survivors formed.

### Telomere Southern blot

Yeast genomic DNA was isolated using a Wizard Genomic DNA Purification Kit (Promega), digested with *Xho*I, separated on a 1% (w/v) agarose gel, and transferred to a Hybond-N+ membrane (GE Healthcare). The membrane was hybridized to a telomere-specific (5′-CACCACACCCACACACCACACCCACA-3′) digoxygenin-labeled probe.

### Measurement of dNTP levels

dNTP levels were measured as previously described (Watt *et al.* 2016).

### Data and reagent availability

Strains are available upon request. The authors affirm that all data necessary for confirming the conclusions of the article are present within the article, figures, and tables.

## Results and Discussion

### Screening for novel genes that are important for type II survivor formation

**Figure 1:**
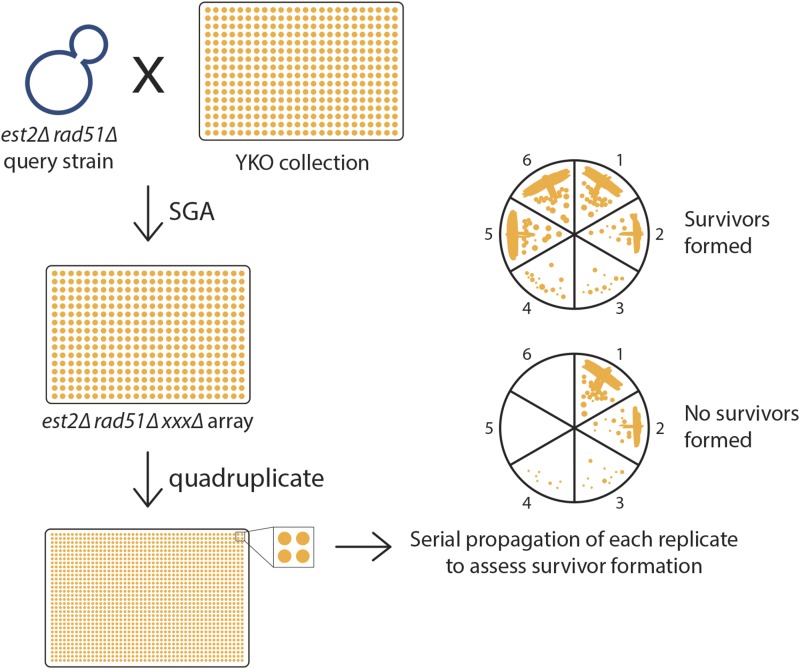
Screening approach for identifying genes important for type II survivor formation. A *MAT*α *est2∆ rad51∆* query strain was crossed to an ordered array of *MAT***a** viable yeast deletion mutants to generate an array of *est2∆ rad51∆ xxx∆* triple mutants via SGA methodology. The triple mutant strains were then quadruplicated by replica-pinning onto fresh agar plates. The resulting four colonies of each *est2∆ rad51∆ xxx∆* triple mutant was then serially propagated up to six times on sectored YPD plates.

To identify genes that are important for type II survivor formation, we screened the yeast knockout (YKO) collection for gene deletions that impair the ability of *est2∆ rad51∆* strains to form type II survivors. We used synthetic genetic array (SGA) methodology (Tong and Boone 2006) to create a library of *MAT***a** *est2∆ rad51∆ xxx∆* mutants, where *xxx∆* is a deletion of a nonessential gene from the YKO collection (Figure 1). Deletion of *RAD51* prevents type I survivor formation (Teng *et al.* 2000; Chen *et al.* 2001), allowing us to screen for genes important for type II survivor formation. Each *est2∆ rad51∆ xxx∆* triple mutant was quadruplicated by replica-pinning, and each replicate was then serially propagated on agar plates to follow senescence and survivor formation (*i.e.*, each *est2∆ rad51∆ xxx∆* strain was tested four times for its ability to form survivors). 32 triple mutants failed to form survivors in all four replicates, 100 failed to form survivors in three of the four replicates, and 403 failed to form survivors in two of the replicates.

All 132 that failed to form survivors in at least three of the four replicates, plus 40 randomly selected that failed to form survivors in two of the four replicates, were further tested by repeating the serial propagation procedure with multiple isolates of single mutants (*est2Δ*), double mutants (*est2Δ rad51Δ*, *est2Δ xxxΔ*, *rad51Δ xxxΔ*) and triple mutants (*est2Δ rad51Δ xxxΔ*) obtained by tetrad dissection of sporulated diploids. This allowed us to compare the phenotypic growth between the selected mutants (*e.g.*, to ensure that loss of viability upon serial propagation was not the result of a synthetic genetic interaction between *rad51∆* and *xxx∆*) and to validate the hits. In this second test, 26 triple mutants failed to form survivors in >50% of the multiple isolates. Only one mutant of these 26 was from the 40 that failed to form survivors in two of four replicates in the original screen, so we did not test any additional genes from this group. Importantly, the 26 included strains with a deletion of *RAD52*, *RAD50*, *RAD59*, *SGS1*, *CLB2*, or *NMD2*, which are all known to be required for type II survivor formation (Lundblad and Blackburn 1993; Teng *et al.* 2000; Chen *et al.* 2001; Huang *et al.* 2001; Johnson *et al.* 2001; Grandin and Charbonneau 2003; Hu *et al.* 2013), as well as *RMI1* and *YLR235C* (which overlaps the *TOP3* open reading frame so that deletion of *YLR235C* likely results in a *top3* hypomorph). Like Sgs1, Top3 is also required for type II survivor formation (Tsai *et al.* 2006). Sgs1, Top3, and Rmi1 form an evolutionarily conserved complex (Chang *et al.* 2005; Mullen *et al.* 2005), so not surprisingly, we find that Rmi1 is also important for type II survivor formation.

To further validate that these genes are important for type II survivor formation, we knocked out each gene in an *est2∆/EST2rad51∆/RAD51* diploid strain of a different genetic background (W303). Once again, we generated haploid meiotic progeny from these diploid strains and serially propagated multiple isolates of each genotype on agar plates to monitor senescence and survivor formation. Overall, 23 genes were identified that are important in type II survivor formation, and of those, 17 genes were not previously reported to be involved in survivor formation (Table 2).

**Table 2:** Genes identified that are important for type II survivor formation

Gene	Fraction of *est2∆ rad51∆ xxx∆* that are able to form survivors		Reference
	in BY4741 background*^a^*	in W303 background	
*CCR4**^b^*		0/10 (0%)	
*CDC55*	0/12 (0%)	2/9 (22%)	
*CHK1*	5/14 (36%)	2/10 (20%)	
*CLB2*	2/14 (14%)		Grandin and Charbonneau 2003
*DOA4*	5/14 (36%)	3/10 (30%)	
*DUN1*	2/12 (17%)	1/25 (4%)	
*LSM1*	5/14 (36%)	0/7 (0%)	
*MOT2*	0/10 (0%)	1/4 (25%)	
*NMD2*	0/12 (0%)		Hu *et al.* 2013
*PPH3*	2/12 (17%)	2/10 (20%)	
*RAD50*	2/10 (20%)		Chen *et al.* 2001
*RAD52*	0/11 (0%)		Lundblad and Blackburn 1993
*RAD59*	4/11 (36%)		Chen *et al.* 2001
*RGI1*	0/4 (0%)	2/10 (20%)	
*RMI1*	1/7 (14%)	0/10 (0%)	
*RPL8B*	1/8 (13%)	2/10 (20%)	
*RPN4*	1/9 (11%)	3/10 (30%)	
*RRM3*	4/12 (33%)	3/10 (30%)	
*SGS1*	0/11 (0%)		Huang *et al.* 2001; Johnson *et al.* 2001
*SPT20*	0/5 (0%)	0/10 (0%)	
*VMA22*	1/10 (10%)	3/10 (30%)	
*YLR235C*	1/16 (6%)	0/10 (0%)	
*YLR358C*	1/5 (20%)	4/9 (44%)	

a These *est2∆ rad51∆ xxx∆* triple mutants were obtained either from the original screen, where four isolates were generated using SGA methodology, or by tetrad dissection of sporulated diploids.

b *CCR4* was not identified in the original screen, but was tested in the W303 background due to its functional connection with *MOT2*.

### Genes involved in the regulation of dNTP pools are important for type II survivor formation

We noticed that two of the identified genes, *DUN1* and *MOT2*, are involved in the regulation of dNTP levels. Dun1 is a DNA damage checkpoint kinase that phosphorylates and inhibits Sml1, Crt1, and Dif1, three negative regulators of ribonucleotide reductase (RNR) (Zhao and Rothstein 2002; Huang *et al.* 1998; Lee *et al.* 2008). The RNR complex catalyzes the rate limiting step in dNTP synthesis (Hofer *et al.* 2012). Mot2 (also known as Not4) is part of the Ccr4-Not complex, a key regulator of eukaryotic gene expression that is required for transcriptional induction of *RNR* genes in response to DNA damage or replication stress (Mulder *et al.* 2005). Ccr4 and Dun1 cooperate to regulate the Crt1-dependent inhibition of the *RNR* genes in response to DNA replication stress (Woolstencroft *et al.* 2006). Although *CCR4* was not identified in our screen, we found that *est2∆ rad51∆ ccr4∆* triple mutants were unable to form survivors (Table 2).

**Figure 2:**
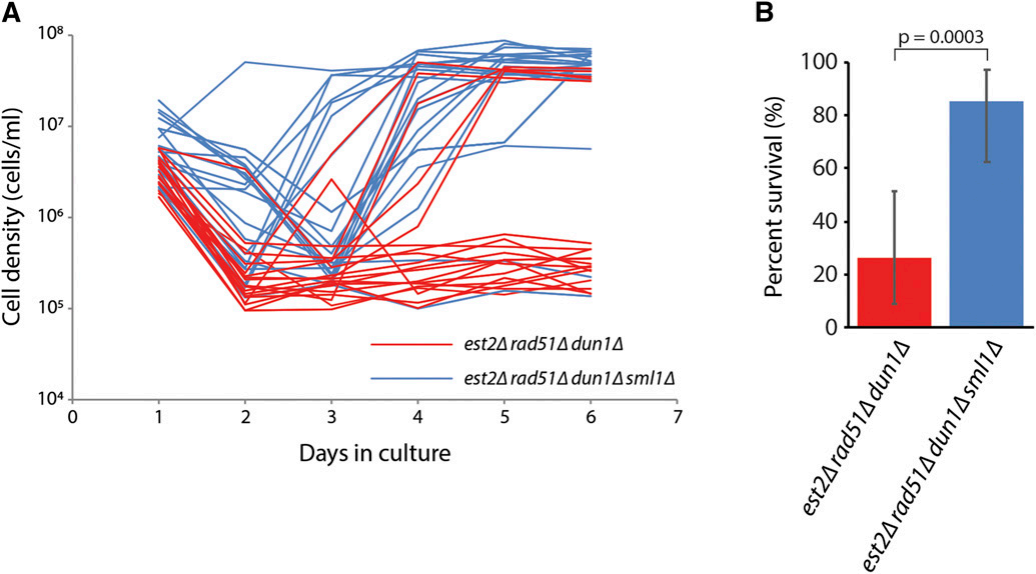
Deletion of *SML1* suppresses the type II survivor formation defect of a *est2∆ rad51∆ dun1∆* strain. (A) Senescence and survivor formation were monitored in liquid culture by serial passaging of individual isolates of *est2∆ rad51∆ dun1∆* (n = 19, red lines) and *est2∆ rad51∆ dun1∆ sml1∆* (n = 20, blue lines), derived from the sporulation of YPM12. (B) Percentage of *est2∆ rad51∆ dun∆* and *est2∆ rad51∆ dun1∆ sml1∆* cultures from panel A that were able to form survivors. Error bars represent exact binomial 95% confidence intervals; p-value was determined using Fisher’s exact test.

The finding that both Dun1 and the Ccr4-Not complex are important for generating type II survivors suggests that the ability to upregulate intracellular dNTP levels is important for the formation of type II survivors. If so, the compromised ability of cells lacking Dun1 or the Ccr4-Not complex to form type II survivors should be suppressed by increasing dNTP levels. To test this hypothesis, we examined whether a deletion of *SML1* could suppress the defect in survivor formation of *est2∆ rad51∆ dun1∆* cells. Sml1 inhibits RNR by binding to Rnr1, the large subunit of RNR (Zhao *et al.* 1998; Chabes *et al.* 1999). Cells lacking Dun1 have a twofold decrease in dNTP levels, but *sml1∆* and *dun1∆ sml1∆* mutants both have a 2.5-fold increase in dNTP levels (Fasullo *et al.* 2010; Zhao *et al.* 1998; Gupta *et al.* 2013). An *est2∆/EST2rad51∆/RAD51dun1∆/DUN1sml1∆/SML1* diploid was sporulated to generate haploid meiotic progeny, which were serially propagated in liquid medium to monitor senescence and survivor formation. We find that deletion of *SML1* largely suppresses the *dun1∆* type II survivor formation defect (Figure 2), suggesting that decreased dNTP levels hinder the formation of type II survivors.

### dNTP pools are upregulated in telomerase-null pre-senescent cells and in type II survivors

**Figure 3:**
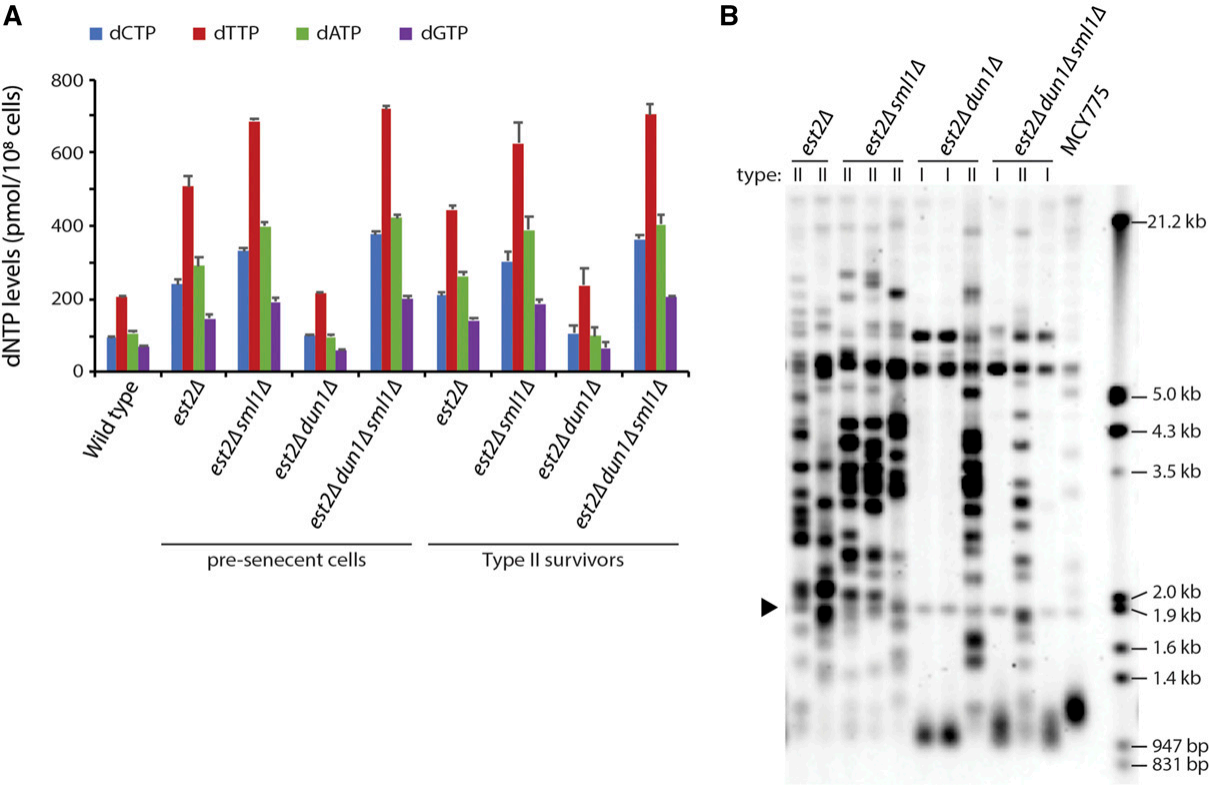
dNTP levels are upregulated in *est2∆* pre-senescent cells and type II survivors. (A) Strains of the indicated genotypes were assayed for dNTP levels. Data are represented as mean ± SE (B) Representative telomere Southern blot of survivors generated by serial propagation in liquid culture of haploid meiotic progeny derived from the sporulation of MCY775. Type I survivors exhibit short telomeres and strong hybridization at 5.2 kb and 6.7 kb due to amplification of the tandemly repeated Y′ short and Y′ long elements, respectively. The telomeres of type II survivors are extended and very heterogeneous in size. The black arrow indicates a 1.8 kb DNA fragment, generated from the BsmAI-digestion of plasmid pYt103 (Shampay *et al.* 1984). This fragment contains telomeric sequences and was ran with each sample as a control.

To confirm our hypothesis that dNTP levels are important for type II survivor formation, we measured dNTP pools in pre-senescent cells (approximately 35 generations after the loss of telomerase) and in type II survivors (Figure 3A). Survivor type was determined by a telomere Southern blot (Figure 3B). We find that dNTP levels are increased in pre-senescent *est2∆* cells and remain elevated in type II survivors. Deletion of *DUN1* abolishes this increase, a phenotype that is suppressed by an additional deletion of *SML1*. These observations suggest that telomere shortening in telomerase-negative cells triggers an increase in dNTP levels that facilitates the generation of type II survivors. Interestingly, an *est2∆ dun1∆* mutant can still form type II survivors, albeit at a reduced efficiency. This indicates that while an increase in dNTP levels promotes the initial formation of type II survivors, it is not needed for maintenance of the survivors.

The elevation in dNTP levels occurs relatively early after telomerase inactivation (ETI; within ∼35 population doublings after the generation of *est2∆* haploid meiotic progeny), well before a majority of cells become senescent. Consistent with this observation, the DNA damage response and expression of *RNR3* is induced in ETI cells (IJpma and Greider 2003; Xie *et al.* 2015). In addition, a recent study has shown that ETI cells experience replication stress, resulting in a dependence on the DNA damage response for viability that is alleviated by elevating dNTP pools via a deletion of *SML1* (Jay *et al.* 2016). Taken together, these findings indicate that replication stress occurs in the absence of telomerase, leading to an upregulation of dNTP levels that promotes the formation of type II survivors. Interestingly, we find that dNTP levels stay elevated in type II survivors (Figure 3), despite these cells looking similar to telomerase-positive wild-type cells in terms of growth rate as well as telomere movement and localization (Teng and Zakian 1999; Straatman and Louis 2007). This observation may be due to the fact that dNTP levels are elevated during BIR (Deem *et al.* 2011), which is required both to prevent accelerated senescence in pre-senescent cells and for telomere elongation in survivors (Fallet *et al.* 2014; Lydeard *et al.* 2007).

In summary, this work has identified novel genes important for the formation of type II survivors. We show that dNTP levels increase early after the loss of telomerase, promoting the formation of type II survivors. However, the increased dNTP levels are not required for the maintenance of type II survivors. Given the similarities between type II survivors and human ALT cancer cells, these findings may help us design more effective strategies to combat cancers that use ALT to maintain telomeres.
